# Ibrutinib Promotes Atrial Fibrillation by Disrupting A-Kinase Anchoring Protein 1-Mediated Mitochondrial Quality Surveillance in Cardiomyocytes

**DOI:** 10.34133/research.0509

**Published:** 2024-10-29

**Authors:** Yukun Li, Xinmeng Liu, Rong Lin, Xiaodong Peng, Xuesi Wang, Fanchao Meng, Shuqi Jin, Wenhe Lv, Xiaoying Liu, Zhuohang Du, Songnan Wen, Rong Bai, Yanfei Ruan, Hao Zhou, Rongjun Zou, Ribo Tang, Nian Liu

**Affiliations:** ^1^ Department of Cardiology, Beijing Anzhen Hospital, Capital Medical University, Beijing 100012, China.; ^2^ National Clinical Research Center for Cardiovascular Diseases, Beijing 100012, China.; ^3^ Department of Cardiovascular Medicine, Mayo Clinic, Scottsdale, AZ 85259, USA.; ^4^ Banner University Medical Center Phoenix, College of Medicine University of Arizona, Phoenix, AZ 85123, USA.; ^5^ Department of Cardiology, Chinese PLA General Hospital, Beijing 100853, China.; ^6^ Xianning Medical College, Hubei University of Science and Technology, Xianning 437000, China.; ^7^ Department of Cardiovascular Surgery, the Second Affiliated Hospital of Guangzhou University of Chinese Medicine, Guangzhou 510120, Guangdong, China.

## Abstract

**Background:** Ibrutinib, a potent Bruton’s tyrosine kinase inhibitor with marked efficacy against hematological malignancies, is associated with the heightened risk of atrial fibrillation (AF). Although ibrutinib-induced AF is linked to enhanced oxidative stress, the underlying mechanisms remain unclear. **Objective:** This research aimed to explore the molecular mechanism and regulatory target in ibrutinib-induced AF. **Methods:** We performed in vivo electrophysiology studies using ibrutinib-treated mice, and then employed proteomic and single-cell transcriptomic analyses to identify the underlying targets and mechanisms. The effects of A-kinase anchoring protein 1 (AKAP1) depletion on mitochondrial quality surveillance (MQS) were evaluated using both in vivo and ex vivo AKAP1 overexpression models. **Results:** Atrial AKAP1 expression was significantly reduced in ibrutinib-treated mice, leading to inducible AF, atrial fibrosis, and mitochondrial fragmentation. These pathological changes were effectively mitigated in an overexpression model of ibrutinib-treated mice injected with an adeno-associated virus carrying Akap1. In ibrutinib-treated atrial myocytes, AKAP1 down-regulation promoted dynamin-related protein 1 (DRP1) translocation into mitochondria by facilitating DRP1 dephosphorylation at Ser637, thereby mediating excessive mitochondrial fission. Impaired MQS was also suggested by defective mitochondrial respiration, mitochondrial metabolic reprogramming, and suppressed mitochondrial biogenesis, accompanied by excessive oxidative stress and inflammatory activation. The ibrutinib-mediated MQS disturbance can be markedly improved with the inducible expression of the AKAP1 lentiviral system. **Conclusions:** Our findings emphasize the key role of AKAP1-mediated MQS disruption in ibrutinib-induced AF, which explains the previously observed reactive oxygen species overproduction. Hence, AKAP1 activation can be employed to prevent and treat ibrutinib-induced AF.

## Introduction

Cancer therapeutics have made remarkable progress over time, significantly prolonging the survival period of cancer patients. However, the accompanying increased risk of cardiovascular diseases has emerged as a crucial factor affecting the prognosis of cancer survivors [1–3]. Anticancer drugs have exhibited considerable cardiotoxicity, which greatly limits their clinical applications. Consequently, cardio-oncology has become a field of common concern for both oncologists and cardiologists. Ibrutinib, an efficacious Bruton’s tyrosine kinase (BTK) inhibitor endorsed by the US Food and Drug Administration for treating chronic lymphocytic leukemia, mantle cell lymphoma, and so on [4,5], is linked to elevated risks of atrial fibrillation (AF) and hemorrhagic complications. A 10-fold increase in the incidence of AF has been reported in the ibrutinib arm [4]. Long-term studies on the use of ibrutinib have revealed AF rates of up to 16% with an average observation period of 28 months [6]. Managing AF associated with ibrutinib use is complex, primarily due to the necessity of balancing the benefits of anticoagulation that it affords against the bleeding risk resulting from its use [7].

Chemotherapy-induced cardiotoxicity involves various mechanisms. Mitochondrial damage is a critical event in cardiotoxicity induced by chemotherapy drugs, such as doxorubicin and sunitinib [8,9]. This damage manifests as imbalanced mitochondrial dynamics, suppressed respiratory function, and so on [10]. Because AF is associated with rapid atrial activation rates that require a high energy supply from mitochondria, metabolic remodeling centered on mitochondrial dysfunction is identified as the chief factor driving the onset and progression of AF [11].

The mitochondrial quality surveillance (MQS) system is closely linked to maintaining mitochondrial homeostasis and restoring any damage to the organelle. The optimal mitochondrial physiological function can be maintained by repairing or removing the damaged mitochondria using regulating MQS factors, such as mitochondrial fission/fusion balance, bioenergetics, and biogenesis. Although studies have explored the role of an impaired MQS system in both cardiovascular diseases and chemotherapy-induced cardiotoxicity like doxorubicin [11–13], the role of an altered MQS system and its upstream regulatory mechanisms in ibrutinib-induced AF have remained largely unexplored. A-kinase anchoring protein 1 (AKAP1) is regarded as a crucial regulator of MQS including mitochondrial metabolism and dynamics. By anchoring the related proteins on the mitochondrial outer membrane (MOM), AKAP1 integrates multiple key pathways to influence mitochondrial morphology, function, and cellular fate. On one hand, AKAP1 promotes mitochondrial respiration and increases the adenosine triphosphate (ATP) synthesis rate by facilitating the phosphorylation of essential mitochondrial proteins like NDUFS4 and cytochrome c oxidase [14–16]. Furthermore, AKAP1 modulates the phosphorylation and dephosphorylation-mediated activation of dynamin-related protein 1 (DRP1), a key protein in mitochondrial fission, through interactions with various binding partners on the MOM, thereby exerting regulatory effects on the balance of mitochondrial dynamics [17].

A previous study found that PI3K-Akt pathway inhibition could cause ibrutinib-induced AF [18]. Additionally, Xiao et al. [19] proposed that the C-terminal Src kinase may be a regulatory target in ibrutinib-induced AF. We have previously identified the pathological phenotypes of calcium dysregulation and structural remodeling in the atrial tissue of mouse model with ibrutinib-induced AF [20]. We further reported excessive production of reactive oxygen species (ROS) and the resulting oxidative stress in atrial myocytes as a potential mechanism for ibrutinib-induced AF [21]. Damaged mitochondria markedly contribute to the generation of ROS, and the disruption of the MQS system is a common toxicological mechanism underlying the cardiotoxicity of anticancer drugs. We herein explore alterations in the atrial MQS system and the related regulatory targets in the pathogenesis of ibrutinib-mediated AF.

## Results

### Ibrutinib increases AF susceptibility and promotes mitochondrial fragmentation

To assess the proarrhythmic potential of ibrutinib, we conducted intraesophageal burst pacing and recorded electrocardiograms (ECGs) in both ibrutinib-treated and control groups. The former exhibited a significantly greater incidence of AF than that of the control group. The duration of burst-pacing-induced AF was longer in the ibrutinib-treated mice (Fig. S1A to C). Echocardiographic evaluation demonstrated that ibrutinib changed the atrial structure. The left atrium (LA) diameter and area of the ibrutinib group were larger than those of the control group (Fig. S1D to F). We further assessed pathological changes in the atria and observed a significant increase in atrial fibrosis in ibrutinib-treated mice (Fig. S1G and H). The ultrastructure of atrial myocytes was observed through transmission electron microscopy (TEM). The mitochondria in the atrial myocytes of the ibrutinib-treated group had a fragmented and punctuated appearance, with significantly shorter lengths than those in the control group, indicating that ibrutinib may disrupt the dynamic balance of atrial mitochondria (Fig. S1I).

### Ibrutinib induces AF by impairing the mitochondrial function and structure of atrial myocytes

**Fig. 1.**
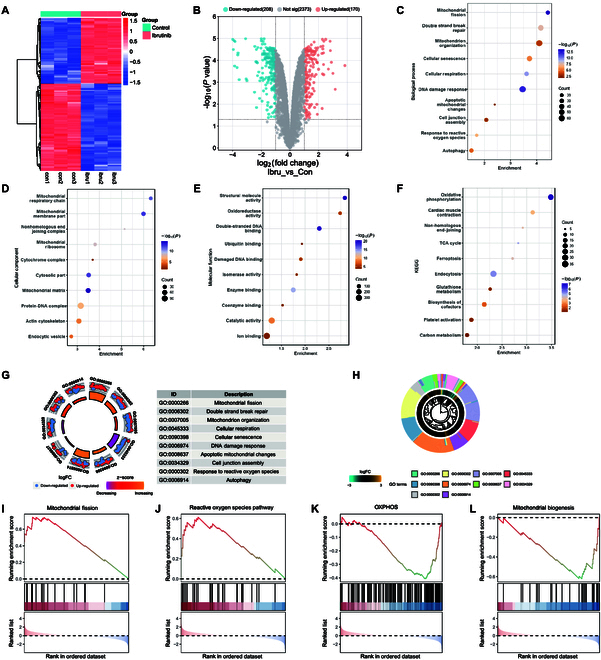
Ibrutinib induces atrial fibrillation (AF) by impairing mitochondrial function and structures of atrial myocytes. (A and B) Expression heatmap and volcano map of differentially expressed atrial proteins, illustrating that 170 proteins were up-regulated and 208 were down-regulated in ibrutinib-treated mice compared with control mice. *N* = 3 independent mice per group. (C to H) Gene Ontology and KEGG enrichment analysis of the 378 differentially expressed proteins in atrial tissue. (I to L) GSEA of differentially expressed proteins following ibrutinib treatment.

We investigated the molecular mechanisms underlying the ibrutinib-induced increased risk of AF. A proteomic analysis was conducted to compare the atrial protein expressions of both the ibrutinib-treated and control groups. The ibrutinib treatment modified the expression of 378 proteins in the murine atrial tissue (|log_2_ fold change| > 1; *P*-adj < 0.05). Of these, 170 proteins were up-regulated and 208 were down-regulated in the ibrutinib group (Fig. 1A and B). A Kyoto Encyclopedia of Genes and Genomes (KEGG) enrichment analysis revealed that mitochondrial metabolic pathways, including oxidative phosphorylation (OXPHOS) and the tricarboxylic acid (TCA) cycle, were significantly altered by the ibrutinib treatment. A Gene Ontology (GO) biological process enrichment analysis showed that the ibrutinib treatment primarily affected proteins involved in the mitochondrial fission, mitochondrial organization, and cellular respiration. Similarly, a GO cellular component enrichment analysis indicated that ibrutinib mainly influenced the proteins located in mitochondrial respiratory chain, mitochondrial membrane, and matrix of mitochondria (Fig. 1C to F). The Gene Set Enrichment Analysis (GSEA) plot demonstrated that ibrutinib up-regulated mitochondrial fission and ROS pathway but impaired OXPHOS and mitochondrial biogenesis (Fig. 1I to L). Hence, ibrutinib intervention appeared to modify the expression of atrial proteins responsible for mitochondrial structure and function.

### Ibrutinib disrupts mitochondrial homeostasis by down-regulating atrial AKAP1 expression

**Fig. 2.**
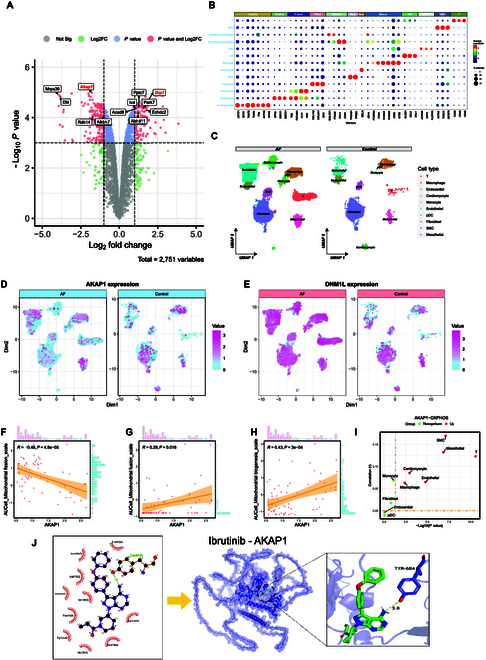
Ibrutinib disrupts mitochondrial homeostasis by down-regulating atrial AKAP1 expression. (A) Volcano plot labeled with the top mitochondria-related differentially expressed proteins. (B) Expression of marker genes for cell-type annotation is indicated on the DotPlot. (C) Uniform manifold approximation and projection representation of all single cells color-coded for their assigned major cell type. (D and E) Featureplots showing the expression patterns of AKAP1 and DRP1 in atrial tissues from AF and control groups. (F to H) Analysis of the correlation between AKAP1 expression and activity score of mitochondria-related pathways. (I) Atrial pan-subpopulation correlation analysis of AKAP1 expression and OXPHOS pathway activity. (J) Molecular docking analysis of the interaction between ibrutinib and AKAP1.

As GO and KEGG enrichment analysis revealed that differentially expressed proteins were primarily associated with mitochondrial structural and functional changes, we annotated highly significant mitochondria-related differentially expressed proteins (−(log_10_ *P*) > 3) in the volcano map. AKAP1 and DRP1 had markedly differential expressions in the ibrutinib group. AKAP1 is crucial for regulating the MQS system. It can maintain the mitochondrial dynamic equilibrium by maintaining DRP1 phosphorylation at Ser637. Hence, off-target effects on AKAP1 could be responsible for ibrutinib-induced AF. Next, we investigated the expression and molecular functions of the aforementioned candidate proteins using single-cell transcriptome data from atrial tissue of patients with AF and control patients. Employing uniform manifold approximation and projection, we performed a hierarchical clustering of cardiac cells from the atrial tissues of patients with the AF and control groups. Sorting the data based on the differential expression of established lineage markers revealed that the atrial tissues comprised 10 major cell types (Fig. 2A to C). The featureplots showed that, in contrast to control samples, atrial tissues from AF patients exhibited decreased expression of AKAP1, especially in the cardiomyocyte subset. Conversely, most cell subsets of the atrial tissue from AF patients displayed increased expression of DRP1 (Fig. 2D and E).

We computed the activity score of the mitochondria-related pathways in the cardiomyocyte subset of the AF group using the AUCell method. The AKAP1 expression was extracted from the AF scRNA-seq dataset, and correlation analysis was conducted with activity scores of mitochondria-related pathways (Fig. 2F to H). The AKAP1 expression was positively correlated with the activities of mitochondrial biogenesis and mitochondrial fusion and negatively with mitochondrial fission. Hence, AKAP1 deficiency could be responsible for excessive mitochondrial fission observed in ibrutinib-treated mice. A pan-subpopulation analysis of the correlation between AKAP1 expression and the OXPHOS activity score in the scRNA-seq dataset of AF patients showed that AKAP1 was significantly associated with mitochondrial metabolism in most atrial subpopulations (Fig. 2I). To further investigate this relationship, we stratified the cardiomyocytes in the subset based on AKAP1 expression. Using the median AKAP1 expression value as a threshold, we categorized the cardiomyocytes into high AKAP1 expression (hiAKAP1) and low AKAP1 expression (loAKAP1) groups (Fig. S2A). Differential expression analysis was performed between these groups, followed by GO and KEGG enrichment analyses. The results revealed that differentially expressed genes were predominantly enriched in biological process terms related to mitochondrial function. These included mitochondrial electron transport, OXPHOS, mitochondrial depolarization, and mitochondrial membrane organization. In terms of cellular process, the genes were enriched in categories such as mitochondrial respiratory chain complex and MOM. KEGG pathway analysis highlighted enrichment in OXPHOS and the TCA cycle (Fig. S2B to E). GSEA provided additional insights. Compared to the hiAKAP1 group, the loAKAP1 cardiomyocyte subgroup exhibited enhanced activation of mitochondrial fission events. Conversely, the OXPHOS pathway, representative of mitochondrial metabolism, was significantly suppressed in the loAKAP1 group (Fig. S2F and G). We performed molecular docking (MD) to elucidate the potential off-target binding interactions between AKAP1 and ibrutinib molecules. The results revealed a binding energy of −6.79 kcal/mol for the protein–ligand complex, suggesting a strong binding effect between ibrutinib and AKAP1 (Fig. 2J).

### AKAP1 down-regulation mediates DRP1 dephosphorylation in HL-1 myocytes

**Fig. 3.**
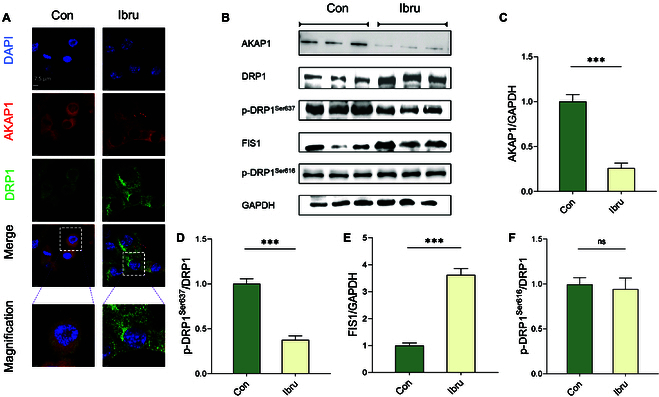
AKAP1 binding mediates DRP1 phosphorylation in atrial myocytes. (A) Immunofluorescence imaging of AKAP1 and DRP1 in ibrutinib-treated atrial myocytes. (B to F) Proteins were isolated from atrial myocytes. Western blotting was used to assess AKAP1, DRP1, FIS1, and phosphorylated DRP1 expression levels. ***P < 0.001. ns, not significant.

We investigated the interaction between AKAP1 and DRP1 and its impact on the mitochondrial dynamic to elucidate the mechanism underlying AKAP1-deficiency-mediated mitochondrial fragmentation. Confocal immunofluorescence imaging demonstrated that ibrutinib treatment decreased the AKAP1 expression (red) and increased the DRP1 expression (green) in HL-1 cells (Fig. 3A). DRP1 phosphorylation is a downstream consequence of the presence of AKAP1 on the outer mitochondrial membrane (OMM). DRP1 possesses an N-terminal guanosine triphosphatase (GTPase) domain. It hydrolyzes guanosine triphosphate to generate the energy necessary for fission. AKAP1, when anchored to protein kinase A, phosphorylates DRP1 at Ser637 within the GTPase effector domain, thus inhibiting its GTPase activity and attenuating mitochondrial fission. A reduction in AKAP1 expression enhances the dephosphorylation of DRP1 at Ser637, facilitating mitochondrial fission. Therefore, we wanted to know whether AKAP1 promotes mitochondrial fission by dephosphorylating DRP1. Western blot analysis revealed that ibrutinib dephosphorylated DRP1 at Ser637 in HL-1 cells, without affecting the phosphorylation levels at Ser616, and simultaneously increased the expression of FIS1, a mitochondrial fission marker (Fig. 3B to F).

### Ibrutinib-induced AKAP1 depletion promotes mitochondrial fission and membrane depolarization via DRP1 dephosphorylation

**Fig. 4.**
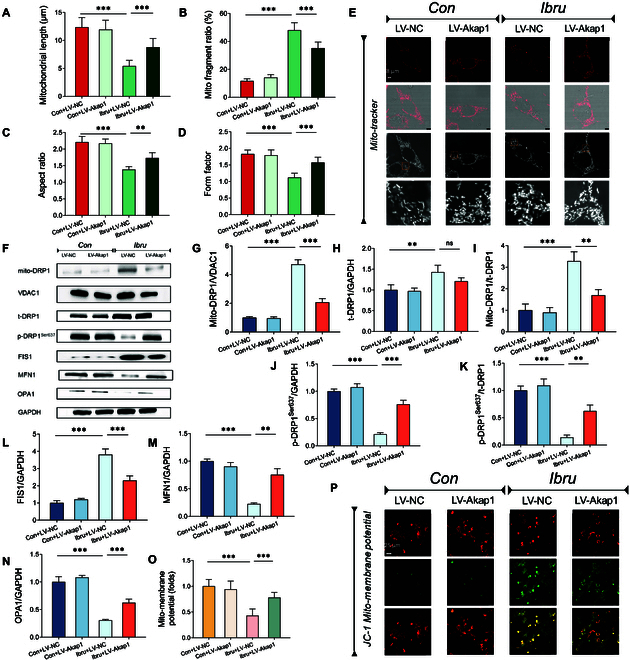
Ibrutinib-induced AKAP1 depletion promotes mitochondrial fission and membrane depolarization via DRP1 dephosphorylation. (A to E) MitoTracker staining for labeling mitochondria in atrial myocytes in vitro. Average mitochondrial length, ratio of fragmented/tubular mitochondria, and mitochondrial morphological parameters were determined. (F to N) Western blot analysis of mitochondrial DRP1 (mito-DRP1), total DRP1 (t-DRP1), phosphorylated DRP1, FIS1, MFN1, and OPA1 in HL-1 cells. VDAC1 was used as a loading control for mitochondrial proteins. (O) Red-to-green ratio of JC-1 fluorescence intensity. (P) Atrial myocytes loaded with JC-1 to analyze changes in mitochondrial membrane potential. *N* = 8 independent cell samples per group. **P < 0.01, ***P < 0.001.

Mitochondrial dynamic imbalance is regarded as both the initiator and facilitator of mitochondrial dysfunction. We quantitatively assessed ibrutinib-induced changes in mitochondrial morphology by considering the fragmented mitochondria in the atrial tissues of ibrutinib-treated mice and the proteomics enrichment analysis results. To further investigate the mechanism underlying AKAP1-deficiency-mediated mitochondrial fission, the HL-1 cardiomyocytes were infected with LV-Akap1 before ibrutinib treatment. MitoTracker staining demonstrated that ibrutinib exposure formed small, round, and fragmented mitochondria in atrial myocytes, indicating enhanced mitochondrial fission. To quantitatively evaluate alterations in atrial mitochondrial fragmentation, we measured the aspect ratio and form factor of mitochondria in both the ibrutinib and control groups. The former displayed a significant decrease in AR and FF parameters than that in the control group. However, transfection with LV-Akap1 partially restored the mitochondrial morphology to normal (Fig. 4A to E).

Western blot analysis showed that ibrutinib exposure led to a significant increase in mitochondrial fission-related proteins (DRP1 and FIS1) and a decrease in mitochondrial fusion-associated proteins (MFN1 and OPA1). Importantly, compared to the modest increase in total DRP1 (t-DRP1), the expression of mitochondria-localized DRP1 (mito-DRP1) was remarkably elevated in the ibrutinib-treated group, along with a significant dephosphorylation of DRP1 at Ser637. Transfecting HL-1 cells with LV-Akap1 considerably increased the phosphorylation level of DRP1 at Ser637. Furthermore, without significant changes in total DRP1, LV-Akap1 transfection diminished DRP1 translocation to mitochondria and enhanced the expressions of MFN1 and OPA1 (Fig. 4F to N). The mitochondrial membrane potential determines the mitochondrial function. Excessive mitochondrial fission can depolarize the mitochondrial membrane potential, which was restored to near-normal levels by AKAP1 overexpression (Fig. 4O and P).

### AKAP1 deficiency impairs mitochondrial respiratory function and promotes metabolic reprogramming in HL-1 myocytes

**Fig. 5.**
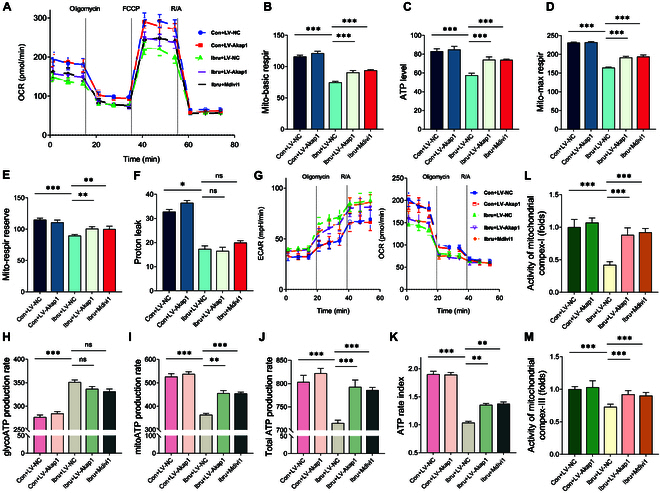
AKAP1 deficiency impairs mitochondrial respiratory function and promotes metabolic reprogramming in atrial myocytes. (A to F) Analysis of HL-1 mitochondrial bioenergetics using the Seahorse XFe96 Analyzer. OCR measurements were taken continuously from baseline and after the sequential addition of 2 mM oligomycin, 1 mM FCCP, and 0.5 mM R/A to measure basal respiration, maximal respiration, spare respiratory capacity, proton leak, and ATP-production levels. (G to J) Total and individual rates of ATP production as mediated by glycolysis or mitochondrial metabolism in HL-1 cells transduced with control and LV-Akap1. (K) Ratio between ATP produced by OXPHOS and that by glycolysis in HL-1 cells transduced with control and LV-Akap1. (L and M) ELISA analysis of mitochondrial respiration complex I/III activities. *N* = 8 independent cell samples per group. *P < 0.05, **P < 0.01, ***P < 0.001.

Mitochondrial bioenergetics and dynamics are interconnected. A disruption in the balance between fusion and fission can impair mitochondrial bioenergetics, diminishing ATP production. We investigated whether ibrutinib disturbs mitochondrial bioenergetics homeostasis by interfering with AKAP1. We conducted a Seahorse Mito Stress test to assess mitochondrial respiration. The results demonstrated that ibrutinib stimulation significantly impaired basal respiration, maximal respiration, spare respiratory capacity, and ATP production, which were attenuated by transduction with LV-Akap1 or cotreatment with Mdivi-1, a mitochondrial fission inhibitor (Fig. 5A to F).

Impaired mitochondrial respiration is linked to metabolic reprogramming. As mitochondrial OXPHOS declines, the real-time ATP rate assay was utilized to assess whether the ibrutinib-treated cells displayed adaptive reprogramming toward the glycolytic pathway. The assay simultaneously measured the basal ATP-production rates from mitochondrial respiration (oxygen consumption rate [OCR]) and glycolysis (extracellular acidification rate). The ibrutinib-treated myocytes had a significantly reduced total ATP-production rate, primarily owing to the inability of the elevated glycoATP rate to compensate for the mitoATP rate deficiency. AKAP1 overexpression or Mdivi-1 cotreatment effectively reversed the metabolic reprogramming phenotype. The degree of this alteration can be determined by the extent of reduction in the ATP index ratio (mitoATP production rate/glycoATP production rate) in ibrutinib-treated myocytes. This reduction was partially restored by AKAP1 overexpression or Mdivi-1 intervention (Fig. 5G to K). These results are consistent with the finding that the activity of mitochondrial ETC complex I/III was compromised in the ibrutinib group but improved to near-normal levels in ibrutinib-treated cells transduced with LV-Akap1 or co-treated with Mdivi-1 (Fig. 5L and M).

### AKAP1 down-regulation is involved in impaired mitochondrial biogenesis, oxidative stress, and inflammation

**Fig. 6.**
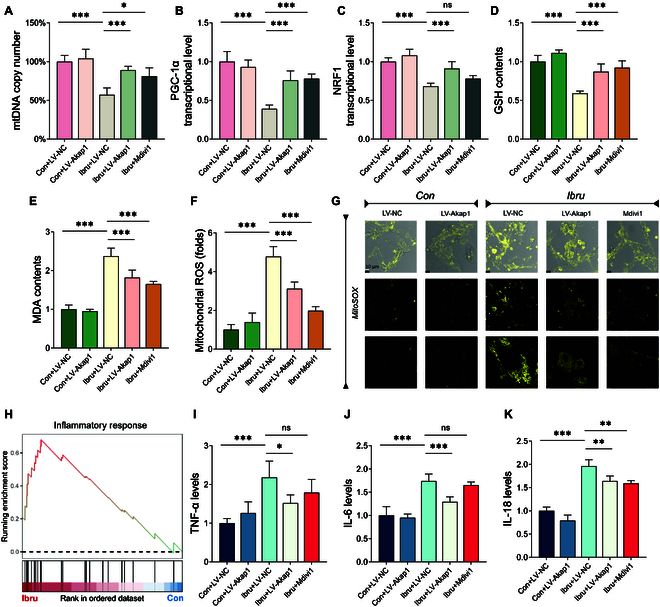
AKAP1 down-regulation in impaired mitochondrial biogenesis, oxidative stress, and inflammation. (A to C) qPCR analysis of mitochondrial biogenesis parameters (mtDNA copy number, PGC-1α, and NRF1). (D and E) ELISA analysis of redox balance biomarkers, including MDA and GSH contents. (F and G) MitoSOX staining of mitochondrial ROS in HL-1 cells. (H) Visual GSEA of inflammation response pathway in ibrutinib-treated myocytes. (I to K) ELISA of atrial inflammatory biomarkers, including TNF-α, IL-6, and IL-18. *N* = 8 independent cell samples per group. *P < 0.05, **P < 0.01, ***P < 0.001.

Insufficient energy production could be associated with defective mitochondrial biogenesis, a process crucial for replenishing number of healthy mitochondria. Ibrutinib treatment reduced the levels of PGC-1 and NRF1, 2 essential regulators of mitochondrial biogenesis. It also reduced the mtDNA copy number. However, this inhibitory effect of ibrutinib on mitochondrial biogenesis was ameliorated by AKAP1 overexpression or Mdivi-1 intervention (Fig. 6A to C).

We next elucidated the regulatory role of AKAP1 in the mitochondrial redox balance in ibrutinib-treated myocytes. Representative MitoSOX staining images showed that ibrutinib promotes excessive mitochondrial ROS production. AKAP1 overexpression significantly diminished the mitoROS level, also observed in the Mdivi-1 cotreatment group. Ibrutinib-treated myocytes exhibited reduced glutathione (GSH) and elevated malondialdehyde (MDA) levels, which were restored by LV-Akap1 transduction and Mdivi-1 intervention (Fig. 6F and G).

Mitochondrial dysfunction not only disturbs energy metabolism but also triggers inflammatory responses through an intricate immunometabolic crosstalk. Given the observed atrial fibrosis in animal models, GSEA of inflammatory response pathway indicated that inflammation activation is presented in ibrutinib-treated myocytes (Fig. 6H). We performed enzyme-linked immunosorbent assay (ELISA) to assess alterations in the levels of inflammatory factors. The ibrutinib group exhibited elevated levels of tumor necrosis factor-α (TNF-α), interleukin-6 (IL-6), and IL-18. However, the AKAP1 overexpression significantly down-regulated these cytokines. IL-18 was reversed in both AKAP1 overexpression and Mdivi-1 cotreatment groups (Fig. 6I to K).

### AKAP1 overexpression prevents ibrutinib-induced AF by improving MQS and restoring mitochondrial function

**Fig. 7.**
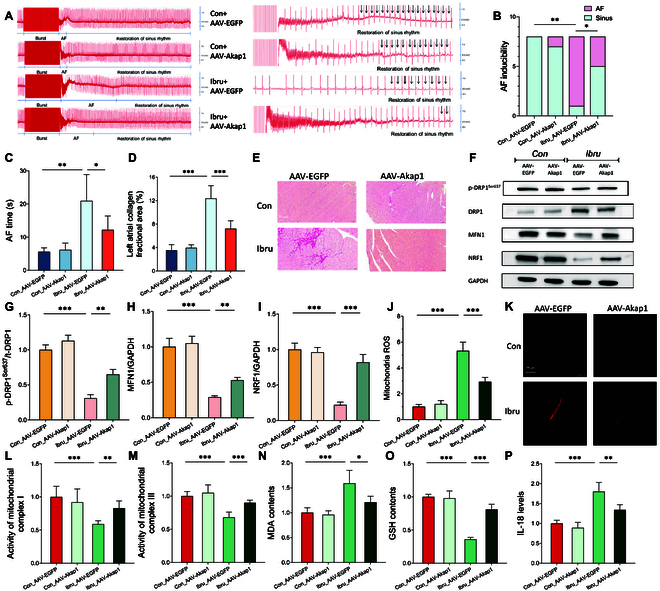
AKAP1 overexpression prevents ibrutinib-induced AF by improving MQS and restoring mitochondrial function. (A) Simultaneous recordings of surface ECG following intraesophageal burst pacing. (B) Quantification of AF time. (C) AF inducibility in control and ibrutinib-treated mice with and without AAV-Akap1 transfection. (D and E) Myocardial fibrosis was assessed in each group through Sirius Red staining. The proportion of fibrotic tissue to myocardial tissue was quantified for each group. (F to I) Western blot analysis of total DRP1 (t-DRP1), phosphorylated DRP1, MFN1, and NRF1 in atrial tissues. (J and K) Images and quantification of isolated atrial myocytes loaded with the mitochondrial ROS indicator MitoSox Red. (L to P) ELISA of MQS/redox/inflammation biomarkers, including complex I/III, MDA, GSH, and IL-18. *N* = 8 mice per group. *P < 0.05, **P < 0.01, ***P < 0.001.

To verify whether AKAP1 up-regulation could mitigate the AF risk and restore the MQS system in ibrutinib-treated mice models, we established an in vivo AKAP1 overexpression model using adeno-associated virus (AAV), with AAV-GFP as the control group. The ibrutinib-treated mice exhibited a higher AF incidence and prolonged AF duration than those of control mice. ECG measurements revealed that the ibrutinib-treated group with AKAP1 overexpression had diminished AF duration and incidence (Fig. 7A to C). AKAP1 overexpression also ameliorated atrial fibrosis (Fig. 7D and E). We validated the ibrutinib-induced Drp1 Ser637 dephosphorylation and expression levels of mitochondrial fission/fusion markers. The ibrutinib treatment reduced DRP1 phosphorylation at Ser637 and simultaneously decreased the MFN1 (fusion marker) and NRF1 (biogenesis marker) levels. These effects were reversed by AKAP1 overexpression (Fig. 7F to I).

MitoSOX staining revealed that mitochondrial ROS production was significantly elevated in ibrutinib-treated mice, which normalized following AKAP1 overexpression (Fig. 7J and K). This finding was supported by the ibrutinib-induced reduction in GSH contents and an increase in MDA contents, which were also reversed by AKAP1 overexpression. Similar trends were observed for the activity of mitochondrial complex I/III. These data confirmed the regulatory role of AKAP1 in mitochondrial bioenergetics in ibrutinib-induced AF. Moreover, the IL-18 level was up-regulated in the ibrutinib-treated group. The AKAP1 overexpression could normalize the inflammatory response (Fig. 7L to P).

## Discussion

**Fig. 8.**
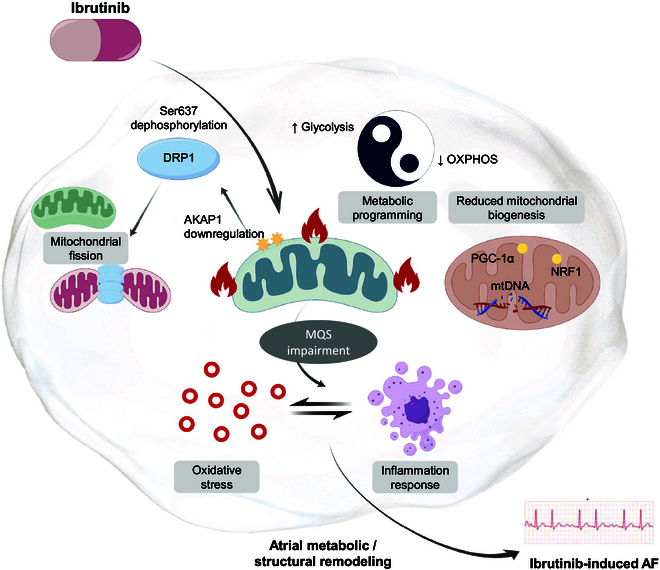
This schematic illustrates the proposed mechanism by which ibrutinib promotes atrial fibrillation (AF). Ibrutinib treatment leads to the downregulation of A-kinase anchoring protein 1 (AKAP1), triggering mitochondrial quality surveillance (MQS) impairment. This results in enhanced translocation of dynamin-related protein 1 (DRP1) to the mitochondria, driving mitochondrial fission. Concurrently, metabolic reprogramming is characterized by increased glycolysis and decreased oxidative phosphorylation (OXPHOS), alongside reduced mitochondrial biogenesis. These mitochondrial dysfunctions elevate oxidative stress and inflammatory responses, contributing to atrial metabolic and structural remodeling, ultimately leading to ibrutinib-induced AF.

Mitochondrial damage serves as a major determinant in the pathogenesis of chemotherapeutic agents’ cardiotoxic effects. We employed gene-editing technologies in animal models and HL-1 cells and showed that ibrutinib increases the AF risk by disrupting the AKAP1-regulated MQS. Our study has several key findings. First, ibrutinib-treated mice exhibited decreased AKAP1 expression, which was closely associated with an increased risk of AF. Second, down-regulated AKAP1 disturbs the MQS system in atrial myocytes, as evidenced by the increased mitochondrial fission caused by enhanced Drp1 Ser637 dephosphorylation and OMM translocation, which is accompanied by impaired mitochondrial biogenesis and mitochondrial metabolic reprogramming in bioenergetics. Third, AKAP1 deficiency in atrial cardiomyocytes dysregulates the MQS, which further promotes oxidative stress and inflammatory activation, resulting in increased atrial fibrosis. Importantly, the AKAP1 overexpression effectively restores mitochondrial homeostasis and alleviates oxidative stress and inflammation-induced fibrosis in the ibrutinib-treated mouse model (Fig. 8). Our results offer novel perspectives on the molecular mechanisms connecting impaired MQS with electrophysiological alterations in ibrutinib-induced AF, indicating that AKAP1 could be a promising target for exploring preventive approaches.

AKAP1, a scaffolding protein, anchors to the OMM with its inserted mitochondrial targeting sequence. By recruiting signaling proteins to the OMM, AKAP1 serves as a mitochondrial signaling hub regulating mitochondrial homeostasis [22,23]. AKAP1 deficiency in retinal ganglion cells causes mitochondrial fragmentation by promoting Drp1 Ser637 dephosphorylation [24]. Previous studies on diabetic cardiomyopathy have revealed that AKAP1 down-regulation can compromise mitochondrial respiration by impeding NDUFS1 translocation into mitochondria and promoting mitochondrial ROS generation [25]. We employed quantitative proteomics to investigate alterations in protein expression following ibrutinib treatment. Enrichment analysis revealed that mitochondria-related pathways were enriched with differentially expressed proteins. The volcano plot showed that AKAP1 was one of the most significantly down-regulated candidates among the mitochondria-related proteins. We validated atrial AKAP1 down-regulation based on AF single-cell transcriptome data. We observed a significant correlation between AKAP1 expression and the activity score of MQS-related pathways, including mitochondrial fission, biogenesis, and OXPHOS in cardiomyocyte subpopulations. Our study is the first to provide a comprehensive understanding of ibrutinib-mediated atrial MQS disturbances through off-target effects.

Recent studies have investigated ROS-related mechanisms in ibrutinib-induced AF. Jiang et al. [20] reported ibrutinib-related atrial calcium handling disorders caused by increased RyR2 Ser2814 phosphorylation. Our recent research has further revealed that enhanced ibrutinib-mediated oxidative stress may increase the Ser2814 phosphorylation of RyR2. This observation is supported by the finding that antioxidant interventions effectively reduced calcium overload and inflammation-induced atrial fibrosis, thereby decreasing the ibrutinib-induced AF risk [21]. During mitochondrial electron transfer, electrons can leak and react with oxygen to create superoxide anions, making mitochondria a major source of ROS. Disruptions in electron transport in damaged mitochondria increase electron leakage, causing ROS bursts. However, as a crucial source of intracellular ROS, the role of mitochondrial damage in ibrutinib-induced AF has not been thoroughly investigated. Through GSEA of proteomics, we found that mitochondrial fission and ROS generation pathways were activated and OXPHOS and mitochondrial biogenesis processes were suppressed in the ibrutinib group. Hence, MQS impairment may be critical for the pathogenesis of ibrutinib-induced AF.

Our study utilized high-throughput sequencing along with genetic editing to investigate the mechanism of ibrutinib-induced mitochondrial damage leading to AF, with a focus on AKAP1-mediated MQS disturbance. The mitochondrial dynamic imbalance is crucial for cardio-oncology. As highly dynamic organelles, mitochondria continuously cycle between fission and fusion events to adapt to environmental demands. During mitochondrial fission, dephosphorylation of DRP1 at Ser637 promotes its recruitment to mitochondria [26]. The recruited DRP1 forms ring-like structures on OMM, constricting the organelle and producing 2 daughter mitochondria. Excessive mitochondrial fission from increased DRP1 Ser616 phosphorylation has also been found to be responsible for the pathogenesis of doxorubicin-induced cardiotoxicity [27].

Excessive fission depletes ATPs by regulating mitochondrial metabolic reprogramming, along with suppressed biogenesis, ultimately disrupting the MQS system. Metabolic reprogramming, a cellular self-regulatory mechanism in response to environmental changes, has been demonstrated to play a crucial role in the pathophysiology of both cardiometabolic and neoplastic diseases [28–31]. This adaptive process has gained increasing recognition and understanding in the field of cardio-oncology, highlighting its role in the complex interplay between cancer therapeutics and cardiovascular health. Sunitinib disturbs mitochondrial bioenergetics by reducing the complex I activities, accompanied by an increased generation of mitochondrial ROS and decreased uptake of substrates, such as nucleosides [32]. A down-regulation of mitochondrial biogenesis markers, including Nrf2, PPARα, and PGC-1α expression, has been reported in the animal models of doxorubicin-induced cardiotoxicity and some other cardiovascular disorders [13,33]. In the mechanism of cardiotoxicity induced by anticancer drugs, the suppression of mitochondrial biogenesis is accompanied by an increase in ROS generation [34]. Interestingly, this dual phenomenon has also been observed in the pathophysiology of AF [35]. This imbalance in mitochondrial homeostasis limits the capacity of atrial energy production and increases ROS generation because of a reduction in the electron transfer efficiency owing to excessive mitochondrial fission in naive daughter mitochondria [36]. The increased ROS levels can attack adjacent intact mitochondria, resulting in a self-perpetuating cycle of ROS-induced ROS production [37]. ROS overproduction and inflammation activation exhibit reciprocal stimulation, creating a vicious loop that forms a pathological substrate for AF.

In this study, we discovered that ibrutinib can disrupt the mitochondrial homeostasis in atrial cardiomyocytes. Indeed, the cardiotoxic side effects exhibited by a series of tyrosine kinase inhibitors are closely associated with the mitochondrial homeostasis disturbance. Sunitinib, a widely used vascular endothelial growth factor receptor tyrosine kinase inhibitor for the treatment of metastatic renal cell carcinoma, has been reported to have common cardiotoxicity with an incidence rate of 1% to 10% [1]. Mechanistic studies have revealed that sunitinib can induce myocardial apoptosis [38], which is manifested as the disruption of mitochondrial network structures [39], reduced efficiency of electron transfer, and the accompanying decrease in ATP production [8]. In H9c2 cardiac cell models exposed to sunitinib for 24 h and mouse models treated by gavage for 2 weeks, the collapse of mitochondrial membrane potential and elevated mitochondrial ROS can be observed. Intervention with mito-TEMPO can effectively alleviate mitochondrial dysfunction. Sorafenib, a multitargeted tyrosine kinase inhibitor, has emerged as a first-line treatment for patients with advanced hepatocellular carcinoma and renal cell carcinoma. Evidence suggests that at the therapeutically relevant doses, sorafenib can directly compromise the mitochondrial function of H9c2 cardiomyocytes [40]. The mechanism underlying this cardiotoxicity may be attributed to sorafenib’s role as an effective ferroptosis inducer [41]. Ferroptosis is typically associated with substantial alterations in mitochondrial morphology, characterized by increased mitochondrial membrane density and reduced mitochondrial cristae [42]. Furthermore, sorafenib has been shown to activate the c-Jun N-terminal kinase downstream pathways, leading to disruption of mitochondrial respiration and induction of apoptosis [43]. Imatinib, which functions through inhibiting the activity of BCR-ABL tyrosine kinase, has become the standard treatment for Philadelphia chromosome-positive chronic myeloid leukemia. However, reports from both in vivo and in vitro studies have highlighted its potential cardiotoxicity. Ultrastructural analysis of cardiac tissue from patients undergoing imatinib therapy has revealed the presence of pleomorphic mitochondria with disrupted cristae. In vitro experiments have provided further insights into the mechanisms of this cardiotoxicity. Cardiomyocytes exposed to imatinib for 24 h demonstrate activation of mitochondrial death pathways, triggered by PERK-eIF2α signaling. This process is accompanied by accumulation of mitochondrial superoxide and disruption of mitochondrial membrane potential [44]. These findings suggest that mitochondrial homeostasis dysregulation may represent a shared pathophysiological mechanism underlying the cardiotoxicity associated with tyrosine kinase inhibitors. This insight opens up new avenues for therapeutic strategies aimed at mitigating the cardiac side effects of these drugs. Future research focusing on maintaining mitochondrial homeostasis could potentially yield promising interventions to reduce the cardiotoxicity of tyrosine kinase inhibitors.

Increasing evidence suggests that targeting mitochondrial homeostasis in the heart could help manage both AF and cardio-oncology. Dipeptidyl peptidase-4 (DPP-4) inhibitors demonstrate upstream preventive effects on AF by enhancing mitochondrial biogenesis [45]. A preclinical study showed that DPP-4 inhibitors can alleviate doxorubicin-induced myocyte deaths [46]. A reduced risk of AF can be observed in patients receiving sodium-glucose co-transporter 2 (SGLT2) inhibitors [47]. The electrophysiological protective actions of SGLT2 inhibitors may be associated with metabolic regulation via AMPK activation, resulting in decreased mitochondrial fission and suppressed ROS generation [48]. Recent research emphasizes the therapeutic promise of SGLT2 inhibitors in cardio-oncology. TriNetX data analysis demonstrated that SGLT2 inhibitors significantly lowered the risk of heart failure worsening and AF in patients with cancer treatment-related cardiac dysfunction [49], which is tied to pharmacological actions of attenuating oxidative stress and stimulating mitochondrial biogenesis [50,51]. AKAP1 overexpression in ibrutinib-treated mice can markedly enhance mitochondrial homeostasis and ultimately decrease the AF risk. Indeed, the AKAP1 overexpression strategy has demonstrated therapeutic benefits by restoring mitochondrial homeostasis in several diseases, including diabetic kidney disease, glaucoma, and diabetic cardiomyopathy [24,25,52]. However, specific pharmacological activators for AKAP1 are not yet available.

### Study limitations

Firstly, although ibrutinib-induced AF has marked clinical implications, ibrutinib-related ventricular arrhythmias should not be overlooked. Future studies need to explore MQS disruption and its regulatory mechanism in both atrial and ventricular myocytes. Secondly, while our study suggests that AKAP1 overexpression may effectively improve mitochondrial homeostasis and reduce the ibrutinib-induced AF risk, there is currently a lack of targeted AKAP1-activating drugs for clinical use. The development potential and pharmacokinetics of AKAP1 activators need further assessment. Lastly, a well-established mouse model of ibrutinib-induced AF was employed. However, given the substantial differences between murine and human AF phenotypes, this model may not fully capture the complete pathogenesis underlying ibrutinib-induced AF. Despite these shortcomings, our study offers novel insights into the role and regulatory mechanisms of atrial MQS impairment in ibrutinib-induced AF.

## Conclusion

This study highlights the crucial role of AKAP1 and its downstream MQS disruption in ibrutinib-induced AF. Ibrutinib disrupts mitochondrial homeostasis in atrial cardiomyocytes by suppressing AKAP1 expression, causing increased mitochondrial fission, down-regulated mitochondrial biogenesis, and mitochondrial metabolic reprogramming in cardiomyocytes. These alterations promote oxidative stress and inflammatory responses, inducing atrial metabolic and structural remodeling and increasing the susceptibility to AF. The AKAP1 overexpression effectively improves mitochondrial homeostasis, alleviating oxidative stress and inflammation responses, thereby lowering the AF risk. Our findings may help in developing BTK inhibitors with reduced electrophysiological side effects and in designing potential metabolic regulation-based strategies for the prevention and management of ibrutinib-induced AF in current clinical practice.

## Materials and Methods

### Animal model

C57BL/6J mice (8 to 10 weeks, weighing 20 to 25 g) were acquired from the Animal Centre at Anzhen Hospital in Beijing, China. The protocols were sanctioned by the Committee for Animal Care and Use of Anzhen Hospital. Following a previously described method, the mice were given daily intraperitoneal doses of ibrutinib at a dose of 30 mg·kg^−1^·day^−1^ and were assessed after 14 days of treatment [21]. Mice that received intraperitoneal injections of phosphate-buffered saline (PBS) served as the vehicle control. The biological function of AKAP1 was investigated via checking the cardiac-specific overexpression of AKAP1 in mice transfected with adeno-associated virus 9 (AAV-9). Purified AAV-9 encoding Akap1 was provided by BrainVTA (Wuhan, China) Co. Ltd. The mice were allocated into 4 different groups: AAV9-cTNT-EGFP-Con, AAV9-cTNT-Akap1-Con, AAV9-cTNT-EGFP-Ibru, and AAV9-cTNT-Akap1-Ibru. Each mouse was intravenously injected with 100 μl of either 5.0 to 6.0 × 10^13^ GC/ml AAV9-cTNT-Akap1 or 5.0 to 6.0 × 10^13^ GC/ml AAV9-cTNT-EGFP in the tail. All mice were maintained in specific pathogen-free environments with a 12-h light/dark cycle, regulated temperature, and humidity. Food and water were available ad libitum throughout the study period.

### Cell culture and lentivirus transfection

For stable AKAP1 overexpression, HL-1 cells were transduced with either a negative control lentivirus (LV-NC) or an Akap1 overexpression lentivirus (LV-Akap1). The Akap1 overexpression lentivirus was designed, synthesized, and sequence-verified by Obio Technology Corp. (Shanghai, China). To mimic ibrutinib exposure on the atrial myocytes, the HL-1 cells were treated with 1 μM of ibrutinib for 24 h before experimental determinations, as detailed in the related prior study [53].

### In vivo electrophysiology study

Electrophysiological studies on the mice were conducted following previously described methods [21]. To be brief, mice were anesthetized via the intraperitoneal injection of 1% pentobarbital sodium. ECGs were recorded from surface limb leads. The electrode was introduced through the esophagus in close proximity to the LA, to capture ECGs with PowerLab and LabChart 7 software. Rapid pacing (10 ms at 30 Hz) was employed to evaluate susceptibility to pacing-induced AF. The AF phenotype was identified by distinguishing it from sinus rhythm phenotype when the ECGs demonstrated an absence of distinct P waves. We used this characteristic to differentiate AF from sinus rhythm. The restoration of sinus rhythm was confirmed by observing the detectable P waves. Duration of AF episodes of each animal was documented.

### Echocardiography

The Vevo 2100 Imaging System (FUJIFILM VisualSonics, Inc.) was employed to assess cardiac structural and functional parameters [21]. Initially, mice from each group were anesthetized using ether. Following effective sedation, the mice were laid in the supine position, their fur was trimmed, and their skin was cleansed before examining the atrial structural indicators. Ultrasound assessments were conducted to measure the LA diameter and area. A technician blinded to the grouping of the mice conducted the echocardiographic examination.

### Atrial histopathology

The left atrial tissue samples were preserved in 4% paraformaldehyde and subsequently sectioned. The sections were stained with Sirius red dye and examined under light microscopy. Hearts from the mice of each group were stained and analyzed. Atrial macroscopic changes were assessed using optical microscopy, with samples observed at a magnification of 200×. Longitudinally cut samples were evaluated for each sample. Quantitative analysis of the images was performed using Image-Pro 6.0 software.

### Transmission electron microscopy

Retrograde aortic perfusion with 2.5% glutaraldehyde was employed to fix left atrial tissue samples from each group. Subsequently, the myocardial tissues of the mice were processed according to standard protocols and examined using TEM as described in our previous study [21]

### Proteomic analysis

The samples were prepared by initially grinding in liquid nitrogen to obtain cell powder, which was then lysed in a buffer of urea and protease inhibitor cocktail. The lysates were sonicated thrice on ice and clarified by centrifugation at 12,000 *g* for 10 min at 4 °C. The bicinchoninic acid assay kit was employed to quantify the protein concentration in the supernatant. For protein digestion, the specimens were treated with 5 mM dithiothreitol at 56°C for a duration of 30 min, followed by alkylation using 11 mM iodoacetamide in darkness at ambient temperature for 15 min. It is followed by an overnight trypsin digestion at a trypsin-to-protein mass ratio of 1:50, with a subsequent digestion at a 1:100 ratio for an additional 4 h.

The resulting peptides were resuspended in 0.1% formic acid and subjected to liquid chromatography–tandem mass spectrometry analysis. The peptides were loaded onto a reversed-phase analytical column and eluted with a gradient of acetonitrile in 0.1% formic acid at a flow rate of 400 nl/min using a NanoElute UPLC system. The eluted peptides underwent ionization and were subsequently examined using a timsTOF Pro mass spectrometer. Data acquisition was dynamically managed to prevent redundant scanning. Mass spectrometry data were processed using MaxQuant (v1.6.6.0) against the Mus_musculus 10090-SP 20190513 database. A reverse library was incorporated for estimating the false discovery rate (FDR) and a common contamination library was employed to filter out contaminants. The FDR thresholds for identifying proteins and peptide-spectrum matches were established at 1%.

Differential analysis was carried out using the limma package. Protein expression heatmaps and volcano plots were generated using freely accessible web-based bioinformatics tools. Mitochondria-related proteins were selected following the MitoCarta 3.0 database. The FunRich database was employed to conduct functional enrichment analyses for biological processes and pathways to categorize differentially expressed proteins. The GSEA utilized GSEA version 4.1.0 from the Broad Institute. A ranked list of differentially expressed proteins between the ibrutinib-treated and control groups was compiled based on their log_2_ fold change values. Gene sets were selected from the BioCarta, Hallmark, Reactome, and MSigDB databases to ensure comprehensive coverage of biological functions and pathways. The permutation test was set to 1,000 iterations, using phenotype permutations to assess the statistical significance of gene set enrichment. FDR correction was applied with a threshold of 0.25 to determine significantly enriched gene sets. Results were visualized using enrichment plots.

### Single-cell RNA-seq analysis

The single-cell transcriptome dataset (GSE161016, PRJNA815461) from the Gene Expression Omnibus database, which includes data from the atrial tissue of healthy controls and patients with persistent AF, was utilized in this study [54,55]. Single-cell RNA-seq data of the atrial appendage tissue from the patients with AF were acquired from PRJNA815461, while that of the atrial tissue from the control group was obtained from 2 patients who had no prior history of coronary artery disease (GSE16101). The Seurat R package was employed to preprocess the raw data. Single cells were filtered (cells expressing a range of 500 to 4,000 unique genes and less than 10% mitochondrial transcripts were retained) for the subsequent analysis. Uniform manifold approximation and projection was used to group and visualize the cells, allowing for a clear differentiation and visualization of cell clusters. Statistically significant cell marker genes (adjusted *P* < 0.05) were identified and utilized to classify the clustered cells into their respective cell types. The FeaturePlot function was then used for gene expression distributions. Furthermore, the R package AUCell was utilized to assess the mitochondria-related pathway activities of specific cells.

### Molecular docking

MD was performed using the AutoDock vina software (Scripps Research, San Diego, CA, USA) to dock proteins and small molecules. The PLIP tool (https://plip-tool.biotec.tu-dresden.de/plip-web/plip/index) was used to analyze the docking results. The 2-dimensional structures from the MD results were depicted with LIGPLOT software v4.5.3 (European Bioinformatics Institute, Cambridge, UK). PyMOL was used to generate the MD maps.

### Confocal imaging

Fluorescent images were obtained with a 40× oil immersion objective on the laser scanning confocal microscope operating in line-scan mode (400 Hz). Cells were plated on gelatin-coated cytospin slides, allowed to air dry, and fixed with paraformaldehyde for 30 min at 20°C. Following a PBS wash, the cells were permeabilized for 15 min with 0.3% Triton X-100 and then blocked at room temperature with 10% bovine serum albumin for 1 h. Then, cells were incubated overnight at 4°C with primary antibodies, followed by the treatment with fluorescence-labeled secondary antibodies for 1 h. The nuclei were stained using 4′,6-diamidino-2-phenylindole. Table S1 lists the antibodies used for immunofluorescence.

MitoTracker Red (0.5 μM, 30 min) was employed to analyze mitochondrial morphology. The form factor and aspect ratio of mitochondria were quantified based on the number of branches, branch junctions, and the total branch length in the mitochondrial skeleton [56]. JC-1 dye (1 μM, 30 min) was employed to evaluate mitochondrial membrane potential, with the ratio of red fluorescence (aggregated) to green fluorescence (monomeric) indicating the mitochondrial membrane potential. Mitochondrial superoxide production was evaluated using the MitoSOX Red (5 μM, 20 min) Reagent.

### ELISA and seahorse assay

ELISA was used to measure the activities of mitochondrial ETC complex I/III, TNF-α, IL-6, IL-18, and the levels of GSH and MDA, following the manufacturer’s protocol (Jiancheng, China). The Seahorse XF96 Extracellular Flux Analyzer was employed to assess mitochondrial bioenergetics when the HL-1 cells reached 95% confluence (5 × 10^3^ cells/well) on microplates. Before the assay, the medium was exchanged with the unbuffered XF medium, and the cells were equilibrated for 1 h at 37 °C in a CO_2_-free environment.

The mitochondria stress test was performed by monitoring the cellular OCR in a seahorse assay medium (catalog no. 103575-100), which was enriched with 1 mM sodium pyruvate, 2 mM glutamine, and 25 mM glucose, with the pH adjusted to 7.4 at 37 °C. Oligomycin at a concentration of 1 μM, carbonyl cyanide 4-(trifluoromethoxy)phenylhydrazone (FCCP) at 1 μM, rotenone at 0.5 μM, and antimycin A (AA) at 0.5 μM were sequentially introduced during the assay. Real-time ATP rate was assessed in the seahorse assay medium containing glutamine (2 mM), glucose (10 mM), and pyruvate (1 mM), with the pH adjusted to 7.4 at 37 °C. The assay was conducted by sequentially adding oligomycin (1.5 μM), rotenone (1 μM), and AA (1 μM).

### Isolation of primary atrial myocytes and atrial mitochondria

Atrial myocytes were isolated using the established enzyme digestion protocol utilizing the Langendorff retrograde perfusion technique [57]. To promote adherence, the isolated atrial myocytes were seeded onto a laminin-coated dish. More detailed methodological information of atrial myocytes isolation can be found in the Supplementary Materials. In this study, mitochondria were isolated from atrial myocytes using a mitochondrial isolation kit (Thermo Fisher Scientific, USA). The first step involved collecting the atrial myocytes and centrifuging them at 700 *g* for 5 min at 4 °C to remove the supernatant. The cell pellet was then washed twice with pre-chilled 1×PBS. Subsequently, the cells were resuspended in mitochondrial isolation buffer and subjected to gentle mechanical disruption on ice to lyse the cells. The lysed suspension underwent differential centrifugation: an initial low-speed spin to remove intact cells and larger debris, followed by a high-speed spin to pellet the mitochondria. The resulting mitochondrial samples were either used immediately or stored at −80 °C until further Western blot analysis. The entire procedure was carried out under cold conditions to ensure the integrity and functionality of the mitochondria. For more detailed information, please refer to the manufacturer’s manual (Thermo Fisher Scientific, USA).

### Western blot analysis

Atrial tissues or cultured cell specimens were analyzed using a previously described Western blot protocol. In brief, the samples were lysed in ice-cold RIPA lysis buffer containing 10 mM of Tris-Cl (pH 8.0), 1 mM of EDTA, 1% Triton X-100, 0.1% sodium deoxycholate, 0.1% SDS, 150 mM of NaCl, 1 mM of phenylmethylsulfonyl fluoride, and 0.02 mg/ml each of aprotinin, leupeptin, and pepstatin. The lysates were sonicated and clarified by centrifugation. Protein concentration was determined using a Bradford assay. Then, sodium dodecyl sulfate–polyacrylamide gel electrophoresis was employed to separate 50 to 100 mg of proteins per lane. The resolved proteins were then transferred to a polyvinylidene difluoride membrane and subsequently incubated with the antibodies documented in Table S1.

### mtDNA copy number and quantitative PCR

The mitochondrial DNA copy number was determined by comparing the cytochrome c oxidase subunit 3 (*Cox3*) mtDNA gene with the UCP2 nuclear gene using quantitative polymerase chain reaction (qPCR). To quantify mtDNA mRNA, Trizol reagent (Life Technologies Inc., Carlsbad, CA, USA) was used to extract total RNA, which was then reverse-transcribed using oligo (dT)-primed cDNA. Table S2 provides the primers utilized for qPCR analysis of the respective sequences.

### Statistical analysis

Data were shown as mean ± standard deviation. Two groups were compared using either the parametric Student’s *t* test or the nonparametric Mann–Whitney test. For comparisons among multiple groups, parametric one-way analysis of variance was employed, followed by the Bonferroni test to determine significance. A *P* value of less than 0.05 was considered statistically significant. R package v4.2 and GraphPad Prism v8.0.2 were used for statistical analysis.

## Data Availability

The data supporting the findings of this study are provided within the main text and Supplementary Materials and can be requested from the corresponding authors upon reasonable request. AF-related scRNA-seq data are available on the National Center for Biotechnology Information Gene Expression Omnibus database.
