# Supplementary figures and images for: Ibrutinib Promotes Atrial Fibrillation by Disrupting A-Kinase Anchoring Protein 1-Mediated Mitochondrial Quality Surveillance in Cardiomyocytes
Source: Research (Wash D C). 2024 Oct 29;7:0509. doi: 10.34133/research.0509 (PMC11518619; doi:10.34133/research.0509)

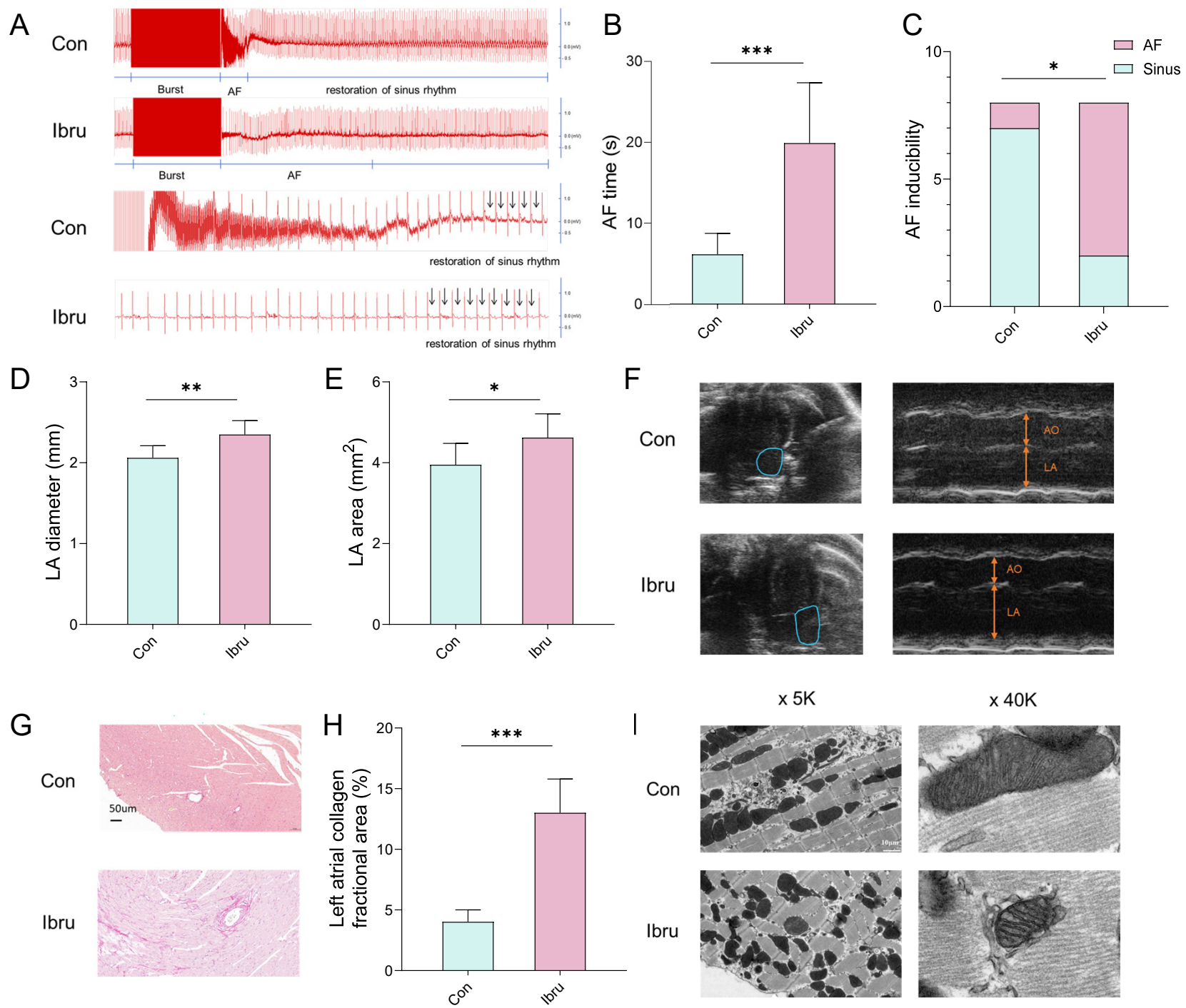

Supplement: Supplementary 1 — Supplementary Text Figs. S1 to S3 Tables S1 and S2 [file research.0509.f1.zip › FigS1.pdf]

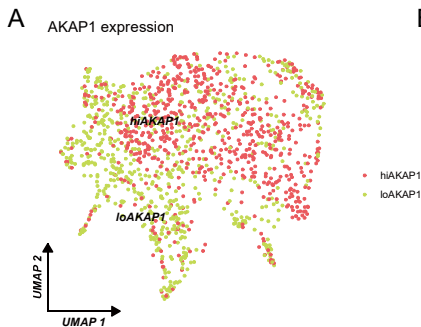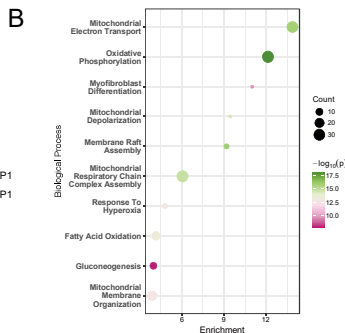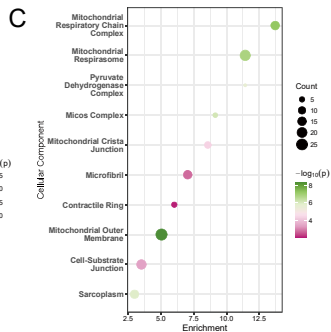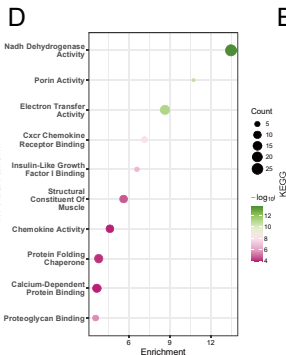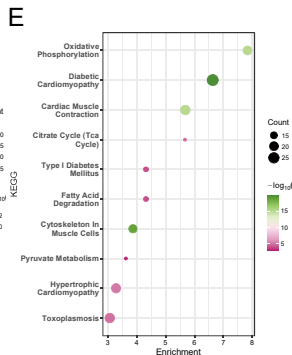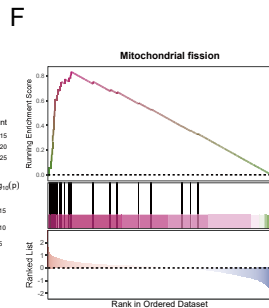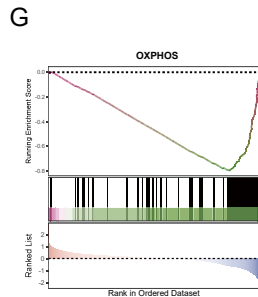

Supplement: Supplementary 1 — Supplementary Text Figs. S1 to S3 Tables S1 and S2 [file research.0509.f1.zip › figS2.pdf]

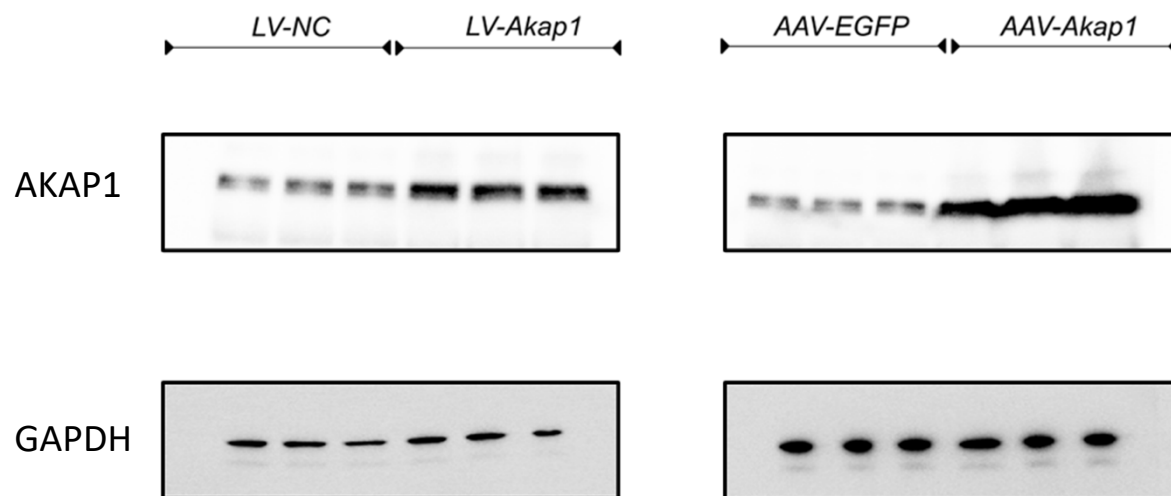

Supplement: Supplementary 1 — Supplementary Text Figs. S1 to S3 Tables S1 and S2 [file research.0509.f1.zip › FigS3.pdf]
