# Supplementary material for: Ibrutinib Promotes Atrial Fibrillation by Disrupting A-Kinase Anchoring Protein 1-Mediated Mitochondrial Quality Surveillance in Cardiomyocytes
Source: Research (Wash D C). 2024 Oct 29;7:0509. doi: 10.34133/research.0509 (PMC11518619; doi:10.34133/research.0509)
Supplement: Supplementary 1 — Supplementary Text Figs. S1 to S3 Tables S1 and S2 [file research.0509.f1.zip › Supplement.doc]

Supplementary Materials and Methods:

1. Isolation of primary atrial myocytes from adult mice

1.1 Preparation of Solutions

Prior to conducting the experiments, it is necessary to prepare five different solutions for the isolation and preservation of the myocytes.

| Solution | Composition (mmol/L) |
| --- | --- |
| Perfusion solution | 113 NaCl, 4.7 KCl, 0.6 KH_2_PO4, 0.6 Na_2_HPO_4_, 1.2 MgSO_4_, 12 NaHCO_3_, 10 KHCO_3_, 10 HEPES, 15 Taurine, 5 Glucose, 10 2,3- butanedione monoxime |
| Digestion solution | 25mL perfusion solution, 25mg collagenase II, 50μL (2.5% 10×) trypsin, 6μL 100mmol/L CaCl_2_, |
| Stop solution 1 | 9mL perfusion solution, 1mL Fetal Bovine Serum (FBS), 4μL 100mmol/L CaCl_2_ |
| Stop solution 2 | 9.5mL perfusion solution, 0.5mL FBS, 4μL 100mmol/L CaCl_2_ |
| Cell resuspension solution | 13mL perfusion solution, 7μL 1mol/L CaCl_2_, Bull Serum Albumin |

1.2 Animal preparation

To prevent blood clotting, heparin (1,000IU/mL, 0.2mL per mouse) was administered via intraperitoneal injection to each mouse. After ten minutes, sodium pentobarbital (50mg/kg, I.P) was injected, rendering the mouse unconscious. Twenty minutes post-heparin injection, cervical dislocation was carried out.

1.3 Removal of the Heart and Cannulation of the Aorta

The thorax was opened by cutting through the rib cage to expose the heart. The pericardium and thymus gland were then removed. Upon locating the aorta and its branches, the ascending aorta was transected, and the heart was immediately excised. Concurrently, retrograde cannulation of the aorta was performed to remove any remaining blood.

1.4 Perfusion and Digestion of the Heart

The heart was perfused with a perfusion solution for 3 minutes to further clear any residual blood from the chambers. Subsequently, a digestion solution was applied to disrupt the connections between the myocytes, converting the tissue into individual cells. The digestion process took approximately 12 minutes, and when the heart appeared swollen and translucent, the atria were harvested and triturated using forceps. Following the collection of the atrial myocytes, the enzymatic activity was neutralized using stop solution 1. The solution was then centrifuged at 192×g for 20 seconds, and the supernatant was discarded. The cell pellet was resuspended in stop solution 2 for subsequent procedures.

1.5 Reintroduction of Calcium and Cell Storage

To gradually acclimate the cells to an extracellular environment containing Ca^2+^, the cells suspended in stop solution 2 were exposed to 100mmol/L CaCl_2_ in a stepwise manner: 5μL, 10μL, 15μL, and 20μL of CaCl2 were added every 5 minutes, respectively (totaling 50μL). The stop solution was then removed and replaced with a cell resuspension solution. It was crucial to maintain an appropriate cell density (for atria, approximately 1~2mL of resuspension solution is suitable). Finally, the cells were stored at 28℃, and experiments were conducted within 6 hours. Our team has previously published a comprehensive protocol for the isolation of atrial myocytes using a modified Langendorff method (reference 26)

Table S1 The information of antibody

| Name | No. | Company |
| --- | --- | --- |
| AKAP1 | PA5-101224 | Thermofisher |
| DRP1 | MA5-26255 | Thermofisher |
| p-DRP1^Ser637^ | #4867 | Cell Signaling Technology |
| p-DRP1^Ser616^ | #3455 | Cell Signaling Technology |
| FIS1 | ab229969 | Abcam |
| GAPDH | ab9485 | Abcam |
| VDAC1 | ab306581 | Abcam |
| MFN1 | ab221661 | Abcam |
| OPA1 | ab42364 | Abcam |
| NRF1 | ab175932 | Abcam |

Table S2 The information of PCR primers

| Gene symbol | Forward primers | Reverse Primers |
| --- | --- | --- |
| *Cox3* | TTTGCAGGATTCTTCTGAGC | TGAGCTCATGTAATTGAAACACC |
| *Ucp2* | ATGGTTGGTTTCAAGGCCACA | TTGGCGGTATCCAGAGGGAA |
| *Pgc-1a* | TATGGAGTGACATAGAGTGTGCT | GTCGCTACACCACTTCAATCC |
| *Nrf1* | AGCACGGAGTGACCCAAAC | AGGATGTCCGAGTCATCATAAGA |
